# Bowel Perforation by Crumpled Paper in a Patient Presenting with Acute Abdominal Pain

**DOI:** 10.4103/1319-3767.45060

**Published:** 2009-01

**Authors:** Alireza Bakhshaeekia, Seyed M.V. Hosseini, Tannaz Razmi, Alireza Shamsaeefar

**Affiliations:** Department of Pediatric Surgery, Shiraz University of Medical Sciences, Shiraz, Iran

**Keywords:** Acute appendicitis, foreign body, bowel perforation

## Abstract

Many of the abdominal foreign bodies are due to accidental ingestion. Our objective in this case report is to emphasize the importance of the enquiry about the foreign body in the differential diagnosis of acute abdominal pain. According to our knowledge, this is the first report of bowel perforation caused by paper ingestion. A 14-year-old boy with abdominal pain underwent exploratory laparotomy and was found to have abdominal pus and ileal perforation. A crumpled paper was found at the site of perforation. Postoperative enquiry revealed that the patient had ingested 10 crumpled papers. We highlight that recording the history is an important aspect in the management of patients with acute abdominal pain and that foreign bodies should be included in its differential diagnosis.

Most abdominal foreign bodies are due to accidental ingestion. Nearly 90% of these pass uneventfully, but few cause obstruction or perforation depending on their structure and size. Soft and pliable bodies are usually not mentioned by patients and sometimes may cause bowel perforation.[1,2] Foreign bodies may be introduced through other routes or percutaneously, and it is important to recognize iatrogenic foreign bodies that may be introduced either deliberately or during surgery.

Our objectives in this case report are to emphasize the importance of recording the history in the differential diagnosis of acute abdominal pain, and in this context, report the first case of bowel perforation caused by paper ingestion. To our knowledge, this is the first such case being reported in literature.

## CASE REPORT

A 14-year-old boy presented with right-sided abdominal pain for 3 days. It had started gradually and was aggravated with motion. The patient had a few episodes of nonbilious vomiting and anorexia. He had no history of other medical problems or medication except, a few analgesic tablets that were used for pain relief in the preceding 3 days. Clinically, he was febrile and had rebound tenderness on abdominal examination. Abdominal x-ray showed generalized gaseous distension.

**Figure 1:**
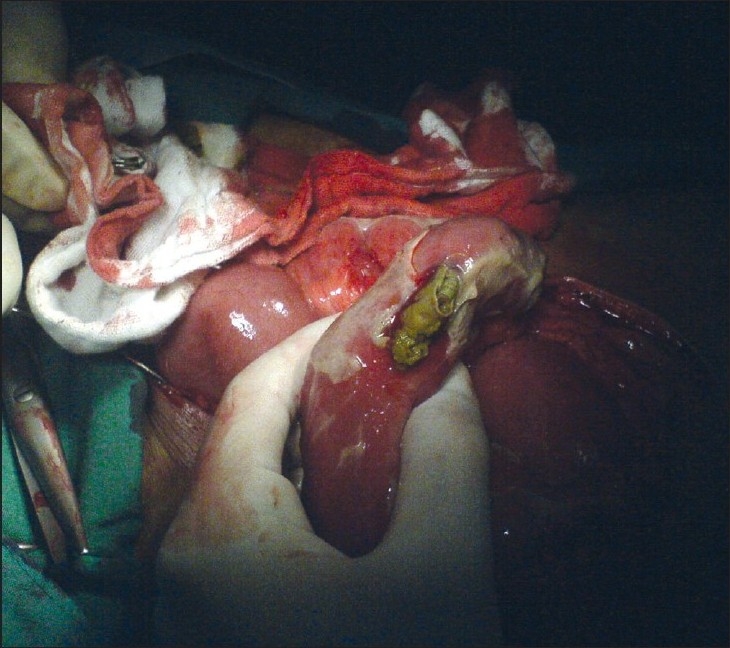
Paper found at the site of intestinal perforation

**Figure 2:**
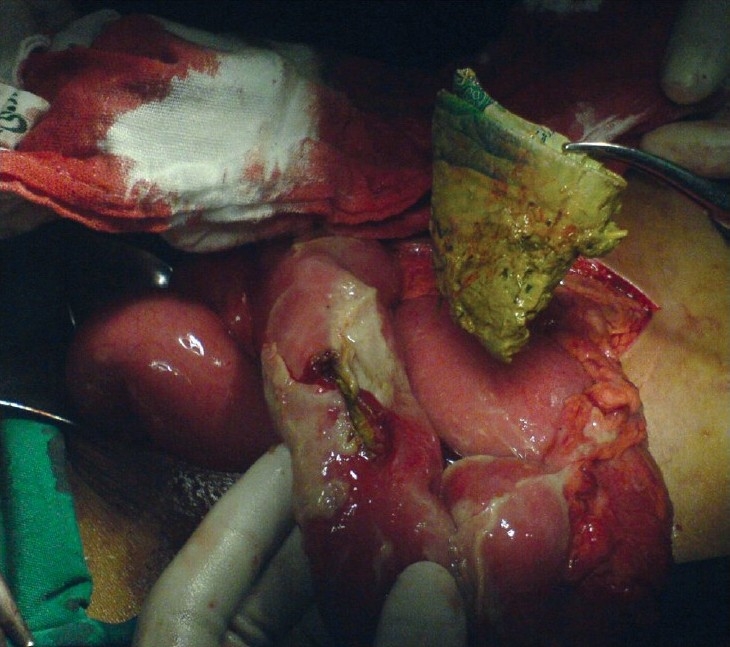
Paper being removed by forceps from the site of intestinal perforation

Patient underwent exploratory laparotomy after an initial working diagnosis of acute appendicitis. Upon exploration, the abdomen was found to contain pus and a 2×1 cm perforation in the 40 cm of terminal ileum with crumpled paper at the site of perforation. Additional undigested papers (5 pieces) were evacuated from the bowel [Figure 1, Figure 2].

Postoperatively, upon enquiry, the patient claimed that he had ingested 10 crumpled papers.

## DISCUSSION

Foreign bodies may sometimes reach the alimentary tract through other routes like the anus, urogenital canal, or percutaneously, either deliberately or by mishap.[3] A detailed history is usually helpful and offers helpful knowledge to the type of foreign body ingested as well as to the expected site of obstruction. Sometimes, history is not available (especially in children, mentally incompetent, or uncooperative patients) or withheld either because of patient embarrassment or to avoid criminal prosecution,[4,5] and 20% of the patients may be asymptomatic.

Whenever one foreign body is known to have been ingested, consideration should be given to the possibility of a second one, and a barium swallow may be required to find these foreign bodies. A barium study is also warranted for follow-up after the acute impaction is resolved, and to detect an underlying stricture that is usually present.[2–6]

A foreign body impacted in the pharynx or esophagus is unlikely to pass spontaneously, and warrants immediate removal.[2,4] The foreign body may stop at the gastric pylorus (thicker than 2.0 cm, longer than 5.0 cm), may fail to traverse the duodenal sweep (longer than 10 cm), or obstruct at the ileocecal valve.[2]

Metal or bony materials are easily seen and lead-containing glass or crystal may be visible in X-ray, but plastic or wood may be challenging to diagnose preoperatively unless there is adherent lead paint. Fish bones and other sharp objects (e.g., other meat bones, toothpicks, razors, and pins) present the risk of perforating the gut wall.[1,7] Sometimes, the penetrating object may seal the perforation and prevent leakage of bowel content until it is removed.[8]

Ideally, sharp objects should be removed while still in the stomach, but round and pliable objects can be followed safely while passing through the alimentary tract, although a few cases of perforation have been reported.[2] However, perforation with paper has never or rarely been reported according to our knowledge.[2]

Metallic foreign bodies require special consideration in their management. The acid of the stomach may react chemically with the metal and result in mucosal inflammation, ulceration, and perforation. Similarly, gastric acid may break the seal of an ingested battery, leading to corrosive toxicity.[6,9–11] The chemical toxicity of heavy metals that may be contained within some of these batteries is also of concern.[6,12,13]

There are few therapeutic options in the management of some of these patients. A bolus may pass spontaneously during a barium swallow, aided by IV glucagon to relieve sphincter spasm, and effervescent agents. Foley's balloon catheter may be introduced although considerable care must be taken with impactions of more than 24-hour duration because of the potential for esophageal perforation from pressure necrosis.[13]

We recommend that the patient's history must be taken precisely to find the cause of acute abdominal pain, and it has considerable impact on the type of management. Foreign body ingestion should be included in the differential diagnosis of abdominal pain and even a soft and pliable object like paper may cause bowel perforation, although it is rarely reported.
